# The tetraspanin Cd63 is required for eye morphogenesis in *Xenopus*

**DOI:** 10.17912/micropub.biology.000335

**Published:** 2020-11-27

**Authors:** Jennifer Kreis, Ramona Bonß, Philipp Vick

**Affiliations:** 1 Institute of Biology, University of Hohenheim, 70599 Stuttgart, Germany

**Figure 1. Loss of Cd63 in the neural ectoderm caused defects in eye morphogenesis:**
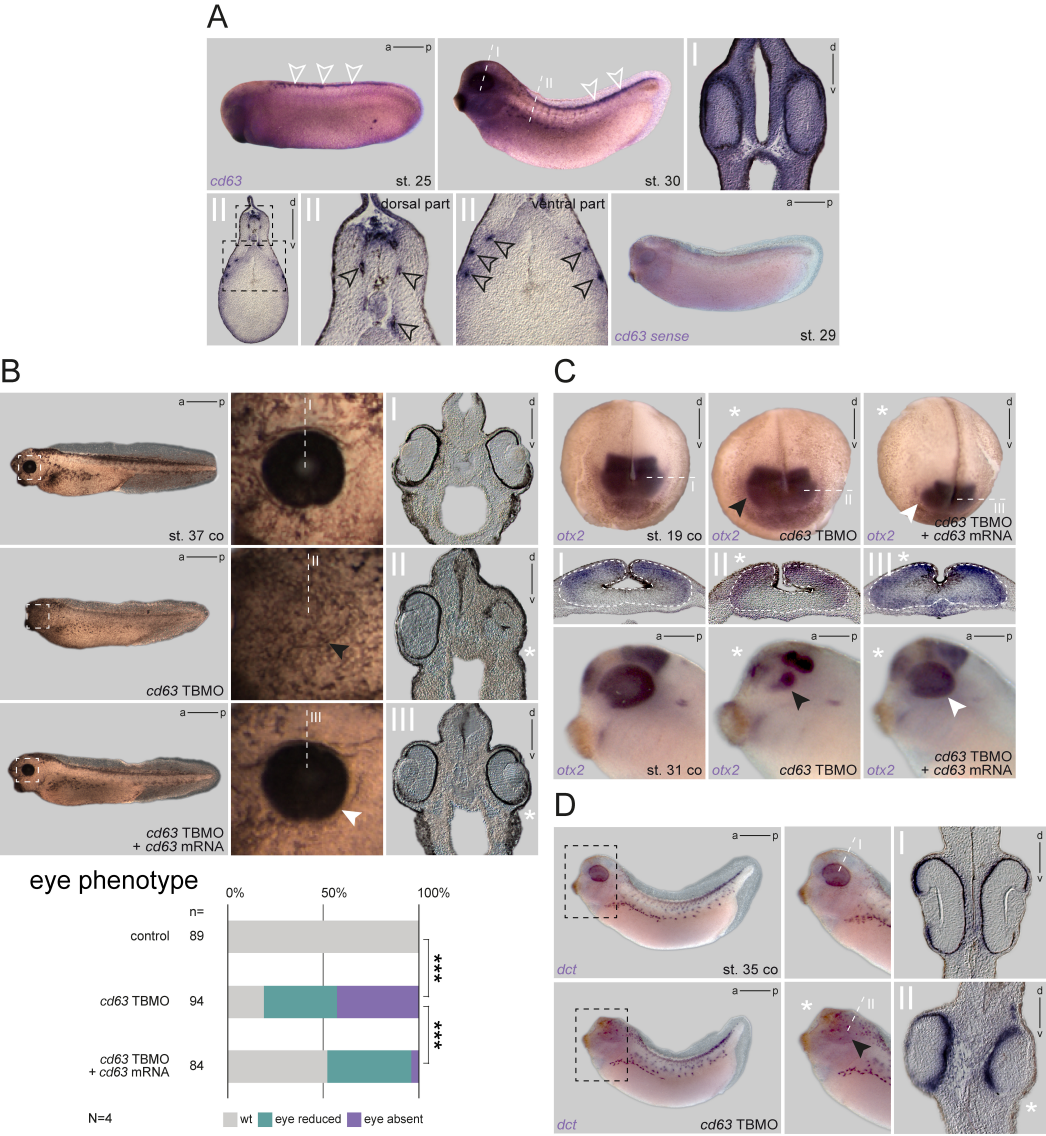
**A** Expression pattern of *cd63* during tailbud stages. Enrichment of transcripts in the TNC of st. 25 (white arrowheads). At st. 30, *cd63* was additionally detected in eyes and cement gland. Transversal sections highlighted signals in the eyes, head mesenchyme, brain, premigratory TNC, dorsal fin mesenchyme, medially and laterally migrating TNC cells (black arrowheads). *cd63* *sense* control specimen of st. 29 did not show any signal. **B** Knockdown of *cd63* in one dorsal animal blastomere resulted in impaired eye development, which was rescued by co-injection of *cd63* mRNA. Sections illustrated normal eye structures in control and rescue specimen, whereas morphant embryos depicted disturbed optic cup formation on the injected side. Quantification of eye phenotypes illustrates significance of the effect. **C** Unilateral loss of *cd63* in the eye field lineage revealed altered expression pattern of *otx2* in neurula and later tailbud stages. At neurulation, *otx2* showed no reduction in intensity but indicated alterations of the targeted eye vesicle (black arrowhead). Sections revealed defects in medial forebrain closure and lateral optic vesicle formation. In tailbud stages, loss of *cd63* resulted in partial reduction of eye tissues (black arrowhead) compared to matching controls. Both effects were reduced by co-injection of *cd63* mRNA (white arrowheads). **D** *cd63* TBMO injection did not block TNC migration or *dct* expression in eyes or melanophores of unilateral morphants. Sections revealed massively impaired formation of eye tissue (black arrowhead) compared to WT eyes of controls. Asterisks mark the injected side. a, anterior; co, control; d, dorsal; p, posterior; TBMO, translation blocking Morpholino oligomer; TNC, trunk neural crest; st., stage; v, ventral; wt, wild type.

## Description

Tetraspanins (TSPAN) are a family of (33 in humans) scaffold proteins consisting of the name-giving four membrane-spanning domains, two extracellular loops, and an intracellular N- and C-terminus. By forming ‘tetraspanin-enriched microdomains’ they participate in organizing the plasma membrane and serve as multipurpose adapters. Some can be found ubiquitously in most cells, while others are specific to certain cell types. On a subcellular level, enrichment in distinct membrane-associated organelles has been documented for several members (Termini and Gillette, 2017).

One member, Cd63, shows such specific enrichment in intraluminal vesicles (ILV) of late endosomes (LE; also called multivesicular bodies, MVB). Further, it is found in exosomes, small extracellular vesicles created from ILV of specialized MVB, which become released after fusion of the MVB with the plasma membrane (Piper and Katzmann, 2007; Pols and Klumperman, 2009; Simons and Raposo, 2009). Another specialized form of LE are melanosomes, which are dedicated for pigment (types of melanin) production in all vertebrates. They are mainly found in two types of tissue, the melanin producing melanocytes (‘melanophores’ in basal vertebrates like amphibians), and in the retinal pigment epithelium (RPE), the outer, light-shielding layer of the vertebrate optic cup (Raposo and Marks, 2007). In amphibians, melanophores derive from trunk neural crest (TNC) cells at the dorsal neural tube, from where they migrate to their lateral, mostly dermal destination during later embryogenesis (Collazo *et al.*, 1993). Cd63 also localizes to early melanosomes, and was shown to be required for melanogenesis in human melanoma cells (Basrur *et al.*, 2003; van Niel *et al.*, 2011).

The externally developing frog *Xenopus* represents a great vertebrate model system for developmental analyses, especially true for the neural crest and its derivatives. However, neither the tissue-specific developmental expression, nor the functional requirement of a specific TSPAN have been tested *in vivo*. Therefore, in this study, we analyzed the expression and function of the *Xenopus cd63* orthologue during tailbud stages, i.e. when major tissue differentiation processes and organ development take place. We were interested if *cd63* showed specific enrichment in melanosome-associated tissues like TNC cells, melanophores or the retinal pigment epithelium (Collazo *et al.*, 1993; Sinn and Wittbrodt, 2013). Further, we tested if loss of *cd63* affected development and function of these tissues.

Expression analysis by *in situ* hybridization (ISH) using a *cd63*-specific probe revealed a striking specific enrichment of mRNA in certain tissues. At early tailbud stages (Figure 1A; st. 25), *cd63* showed strong expression in premigratory TNC cells. This tissue was still positive at later tailbud stage, then localized mRNA signals were also found in the cement gland, head mesenchyme, brain, dermis, dorsal fin mesenchyme, migrating TNC cells and eyes, including the RPE (Figure 1A; st. 30). At this stage, transcript enrichment could also be detected laterally in putative melanophores, i.e. before start of pigment synthesis (Figure 1A). A sense probe control did not detect any signal in these tissues (Figure 1A). These analyses demonstrated that *cd63* was expressed in a tissue-specific manner, indicating a spatial requirement for certain embryonic processes. Moreover, the mRNA distribution suggested a potential role in pigment-associated tissues, i.e. melanophores and RPE.

Thus, we next wanted to test a potential functional requirement of Cd63 for melanogenesis in tadpoles. We designed a translation-blocking morpholino oligomer (TBMO) that binds the 5’UTR of both homeologs of the *X. laevis* *cd63* mRNA, to block protein synthesis. Embryos were injected unilaterally into the neural lineage to target the eyes, TNC, and melanophores. When control embryos or internal control side of morphants showed normal eye and melanophore formation, the targeted side of morphants displayed strong defects in eye morphology (Figure 1B). Most morphant halves displayed reduced or lacking eyes when analyzed externally. Transversal sections confirmed defects in optic cup formation, with defects ranging from partial to nearly complete lack of most major structures (RPE, retina and lens). This effect was partially rescued by co-injection of wild type (WT) *cd63* mRNA lacking the 5’UTR, i.e. insensitive to the MO, demonstrating specificity of the observed phenotype. Rescued halves showed WT or milder phenotypes (Figure 1B). Interestingly, no obvious phenotype was detected in morphant melanophores. Migration appeared not to be altered, and neither number, nor the shape of melanophores was significantly different from control halves. To understand the temporal basis of this eye phenotype, a subset of injected specimens was fixed at late neurula or tailbud stages to check for expression of *otx2*, a forebrain marker highlighting eye tissues (optic vesicle/cup or differentiated eyes, respectively). At neurulation, expression was not reduced after *cd63* knockdown but revealed alterations in prosencephalon shapes on the injected side, indicating a morphogenetic effect (Figure 1C). Transversal sections of such embryos revealed an inhibition of medial closure of the prosencephalon, and impairment of optic vesicle formation (sections I-III in 1C), together causing widening of the *otx2* expression domain on the injected half. Later, when embryos reached tailbud stages, this morphogenetic phenotype appeared more pronounced, resulting in partial reduction of optic *otx2* expression. Again, the severity of both phenotypes was milder in rescued embryos.

Finally, some embryos were checked for expression of *dopachrome tautomerase* (*dct*), a gene coding for a tyrosinase-related enzyme participating in melanin synthesis in the RPE and in melanocytes, and which is highly expressed in these tissues in *Xenopus* as well (Kumasaka *et al.*, 2003). As reported before, expression was highly reminiscent of *cd63* at tailbud stages (cf. Figure 1A). We did not detect any effect on TNC formation, number or migration ability of melanophore precursors, or expression intensity, indicating correct specification of melanophores (Figure 1D). Using *dct* to highlight the RPE, embryos showed again a clear reduction in optic tissues, though, sections revealed remnant RPE expression even in severely affected specimen. Thus, knockdown of *cd63* did not affect melanophore development in our approach. Altogether, from these analyses we conclude that Cd63 is required for correct eye morphogenesis, but surprisingly dispensable for melanophore specification and melanogenesis in *Xenopus*.

While the observed phenotypes were shown to be specific to the loss of *cd63*, we cannot exclude a compensatory effect taking place in the melanophore lineage. In some cases, including Cd63-dependent melanosome formation in mice, it has been shown that the RPE is more sensitive to alterations in the process of melanogenesis as compared to melanocytes, as this process is much more dynamic in the latter (cf. Lopes *et al.*, 2007; van Niel *et al.*, 2011). Nearly no TSPAN gene has ever been analyzed in *Xenopus* development so far; therefore, the loss of *cd63* in melanophores might also be compensated by other TSPAN potentially expressed in these cells as well. Alternatively, in contrast to mouse melanocytes, Cd63 might not directly participate in amphibian melanogenesis (van Niel *et al.*, 2011). It could participate in other cellular processes, not directly related to melanin production, e.g. regulation of exosomes. The lack of impact on migration of TNC derivatives is in agreement with another known role of Cd63, namely its inhibitory effect on cell migration. While known as a factor elevated in early stage melanomas, it has been shown to be a negative driver of advanced stages by inhibiting epithelial-mesenchymal transition, and thus invasive behavior (Radford *et al.*, 1997; Jang and Lee, 2003; Lupia *et al.*, 2014). Therefore, in our knockdown approach, migration behavior of TNC should rather be promoted. Finally, the role of Cd63 in cell migration is tightly associated with its regulatory function of integrin abundance at the plasma membrane, an influence on cell adhesion shared with other TSPAN (Termini and Gillette, 2017). Concerning the morphogenetic phenotypes we observed in the early tadpole eyes, it is interesting to note that correct integrin localization has been demonstrated to be essential for eye morphogenesis in fish, providing an interesting link to our observations (Martinez-Morales *et al.*, 2009).

## Methods

***Xenopus laevis* care and maintenance**

Frogs were purchased from Nasco (Fort Atkinson, WI, USA). Handling, care and experimental manipulations of animals was approved by the Regional Government Stuttgart, Germany (V349/18ZO ‘*Xenopus* Embryonen in der Forschung’), according to German regulations and laws (§6, article 1, sentence 2, nr. 4 of the animal protection act). Female frogs (between 4 and 12 years old) were injected subcutaneously with 300-700 units of human chorionic gonadotropin (Sigma), depending on weight and age, to induce ovulation. Sperm of male frogs was obtained by maceration of testes (stored at 4°C in 1x Modified Barth`s saline with HEPES). Embryos were staged according to Nieuwkoop and Faber (1994). Only clutches of embryos from healthy females were used for the experiments and early embryonic stages were chosen only if survival rates were normal. For experiments, individual embryos from one batch were randomly picked and used as control or tested specimens. If control groups displayed unusual or high percentage of developmental defects later in development, such clutches and experiments were excluded as well, based on empirical judgement.

**Morpholino design, mRNA synthesis and microinjections**

The *cd63* TBMO (sequence: 5’TTCTCCTCTCCAGTAACTTGTAACG 3’) targeting the 5’UTR of both *X. laevis* homeologs was designed using the S-sequence (5’UTR position -40 to -16 distance from the start), as deposited in gene bank (accession: NM_001087051). For rescue experiments, RFP-*cd63*/CS2 plasmid (Lee *et al.*, 2007) containing the *cd63* coding sequence without 5’UTR was linearized with NotI and transcribed *in vitro* (Sp6 polymerase) using Ambion message machine kit. Drop size was calibrated to four nl per injection. One pmol of MO was injected unilaterally into the animal hemisphere at four cell stages with or without rescue mRNA (260 pg per injection), targeting predominantly the neural half of the embryo. The opposite side was used as an internal control.

***In situ* Hybridization**

Partial ISH probes for *X. laevis* *cd63* (accession: NM_001087051) and *dct* (accession: BC073623.1) were cloned using standard RT-PCR with the following primer pairs:

(F) 5’-TTCTTCAACTTCGTGTTCTGG-3’/(R) 5’-CCGTGATATTACTTGTGTTGC-3’ (*cd63*), and

(F) 5’-GCCGCTGAAGTTCTTTAACTC-3’/(R) 5’-GGTAAAGGTAGCATTCATCAAGG-3’ (*dct*).

For *in situ* mRNA detection, ISH was performed after fixation in MEMFA for 2-3h at room temperature and processed following a standard protocols (Sive *et al.*, 2000). RNA *in situ* probes were transcribed using SP6 or T7 polymerases.

**Embryo sections**

For vibratome sections (thickness: 30-35 μm), embryos were embedded in a glutaraldehyde-cross-linked gelatin-albumin mix (Embedding medium: 2.2 g gelatine,135 g bovine serum albumin, 90 g sucrose dissolved in 450 ml PBS) and razor blade-sectioned as indicated in whole-mount panels using a Leica VT1000S vibratome.

**Photo Documentation**

Pictures were taken with a Zeiss SteREO Discovery.V12 (for whole embryos) or an Axioplan2 inverted (for sections) microscope using AxioVision 4.6. Adobe Photoshop CS6 was used for cropping and careful brightness adjustments. All figures were arranged using Adobe Illustrator CS6.

**Statistical analysis**

Statistical calculations of marker gene expression were performed using Pearson’s chi-square test. *=p<0.05, **=p<0.01, ***=p<0.001 were used for all statistical analyses. ‘N’ represents the number of experiments (i.e. number of biological replicates of batches of embryos from different fertilizations), and ‘n’ the number of embryos analyzed (i.e. number of biological replicates of embryos).
